# New Drugs for Obesity, Is the Excitement Affordable?

**DOI:** 10.1089/pop.2023.0086

**Published:** 2023-10-10

**Authors:** Ian Duncan, David Kerr, Rajesh Aggarwal, Nhan Huynh

**Affiliations:** ^1^ Department of Statistics and Applied Probability, University of California Santa Barbara, Santa Barbara, California, USA.; ^2^ Diabetes Technology Society, Santa Barbara, California, USA.; ^3^ Twenty 30 Health, Erdenheim, Pennsylvania, USA.; ^4^ Santa Barbara Actuaries, Inc., Santa Barbara, California, USA.

In recent weeks, sections of the press, social media, and celebrities have been gushing in their praise of a new class of drugs for the treatment of obesity (known as Glucagon Like Peptide-1 Receptor Agonist [GLP-1 RA] eg, Semaglutide with the brand name Wegovy). The drugs are undoubtedly effective in promoting weight loss,^1^ but there has been very little discussion about the cost of the drugs, particularly for payers in the US health system. There are currently no cheaper generic versions and Wegovy is not covered by most Medicare and Commercial insurance plans in the United States.

A recent Economist article (March 3, 2023) cited research that claims that the cost of excess weight from associated comorbidities could amount to 4 trillion dollars, or 2.9% of global GDP.^2^ It would seem, therefore, that a drug that results in significant weight loss would pay for itself. However, a payer will require evidence that such a reduction in weight would result in a sufficient lowering of their medical costs to offset the price of the drug and that this effect is durable.

To avoid complications from the COVID pandemic, we analyzed the 2019 costs of US Commercially insured patients at different levels of obesity (body mass index [BMI]) in the Merative data set, a widely used source of Commercially insured payer utilization and cost data.^3^

Figure 1 summarizes the total cost of care per patient per month for patients with different categories of obesity and other chronic conditions—defined as conditions that would potentially change the way a physician treats obesity, the specific drugs prescribed, and the dosage. Major conditions analyzed were diabetes, cardiovascular disease, renal and behavioral conditions (patients with a single condition in addition to obesity only) as well as for those with 2 or more conditions in addition to obesity.

**FIG. 1.**
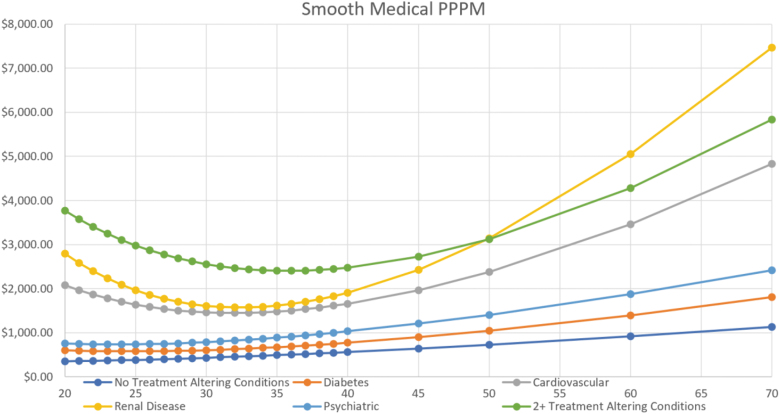
Average allowed charges per patient per month by obesity category and associated conditions.

Individuals with no condition, diabetes, and behavioral conditions had a secular increase in cost as the degree of obesity increased, although at relatively low levels. Increase in cost per unit of BMI between BMI values of 30 and 60 kg/m^2^ were 2.5%, 2.8%, and 2.9% for no conditions, diabetes, and behavioral conditions, respectively. Costs for other conditions (cardiovascular, renal, and 2 or more conditions) declined or were flat in the BMI range 30–40 kg/m^2^.

Although health care costs increased slightly in the BMI range of 30–40 kg/m^2^, they only began to escalate significantly at BMI >40 kg/m^2^.

From our data, for an individual with diabetes and a BMI of 45, the costs averaged $10,800 per year. Reducing weight by 15%–20% (or 7–9 points of BMI) would lower costs to $8200–$8700 per year, an expected reduction of $2000 and $2500 per year. Those with higher costs due to renal disease could expect to see a reduction from about $30,000 annually to $20–$21,000 or a reduction of $9–$10,000 annually. Offsetting the limited reduction in health care costs is the significant increase in costs to payers due to the price of the drug ($12,000–$14,000 annually before discounts and rebates).

Furthermore, for an employer, receiving a 50% reduction in drug price due to discounts and rebates, the treatment would be cost-effective for a member with renal disease but not for an individual with diabetes as a comorbid condition. A limitation of our analysis is the effect of these drugs over time. Currently, data from trials of GLP-RAs are available for 72 weeks and the risk of regaining weight after cessation of treatment^4^ would likely have a further negative effect on the health economics.

In conclusion, given the published weight reduction in the range of 15%–20% with these drugs, there is insufficient reduction in their health care costs for individuals with a baseline BMI between 30 and 40 kg/m^2^. Therefore, we conclude from this analysis that there is no economic case for a payer to reimburse the cost of the drug at the current price point, except in rare cases of very high BMI or certain comorbidities. The question is whether the demand for these drugs will be palatable to insurers and employers?

## References

[1] Bergmann NC, Davies MJ, Lingvay I, et al. Semaglutide for the treatment of overweight and obesity: A review. Diabetes Obes Metab 2023;25(1):18–35. 36254579 10.1111/dom.14863 PMC10092086

[2] World Obesity Atlas 2022. World_Obesity_Atlas_2022.pdf Accessed March 7, 2023.

[3] Real world evidence. Merative. www.merative.com/real-world-evidence Accessed April 9, 2023.

[4] Wilding JPH, Batterham RL, Davies M, et al; STEP 1 Study Group. Weight regain and cardiometabolic effects after withdrawal of semaglutide: The STEP 1 trial extension. Diabetes Obes Metab 2022;24(8):1553–1564. 35441470 10.1111/dom.14725 PMC9542252

